# Evidence of Sertoli cell lineage contribution to the rete testis population during embryonic development

**DOI:** 10.3389/fcell.2026.1849381

**Published:** 2026-06-05

**Authors:** V. V. Mun, M. S. Sabirov, R. H. Rakhimova, E. A. Malolina, A. Y. Kulibin

**Affiliations:** N.K. Koltzov Institute of Developmental Biology, Russian Academy of Sciences, Moscow, Russia

**Keywords:** cell-cell communication, organotypic culture, PAX8, rete testis, RNA velocity, single-cell RNA sequencing

## Abstract

The rete testis (RT) develops at the gonad-mesonephros interface and links seminiferous tubules to efferent ductules, but the cellular origins of RT cells and the mechanisms driving RT expansion during embryonic development remain incompletely resolved. Here, to test whether gonadal Sertoli cell (SC) lineage cells contribute to the developing RT, we applied a chimeric organotypic culture approach combining gonadal and mesonephric compartments of GFP+ and GFP-mouse embryonic urogenital complexes of embryonic day 12.5 (E12.5). The gonadal compartments were dissected to be devoid of PAX8+ RT cells, whereas the mesonephric compartments contained distal RT structures adjacent to mesonephric tubules. After 3 days of culture, newly formed PAX8+ RT cells and continuous RT-like structures were detected in the gonadal compartments. A subset of these PAX8+ cells was localized among SCs within testis cords and co-expressed the SC marker AMH, suggesting that the SC lineage may contribute to the forming RT. When larger portions of the distal RT were retained in the mesonephric compartments, the numbers of newly formed PAX8+ cells in the gonadal compartments increased, indicating that local signaling plays a role in RT formation. In parallel, we reanalyzed a published single-cell RNA sequencing dataset of embryonic mouse testes by focused reclustering of the male supporting lineage (pre-Sertoli, SC, and RT populations), followed by RNA velocity and ligand-receptor-based inference of intercellular communication. Transcriptomic reanalysis identified C5 and SC-Lhx9 clusters with mixed pre-Sertoli, SC, and RT-associated marker profiles. RNA velocity predicted transitions from these clusters toward the RT lineage, and ligand-receptor inference indicated active signaling interactions between these clusters and the RT. In addition, quantitative analysis of RT expansion on E12.5-E16.5 showed that proliferation of resident RT cells alone cannot account for the observed increase in RT cell numbers, which supports the hypothesis that additional cells were recruited. Together, these data support a model in which a subset of proximal RT cells adjacent to testis cords may arise from the SC lineage, and proximal RT formation is promoted by signals from pre-existing distal RT structures. This refines current models of RT morphogenesis and clarifies cellular plasticity at the SC-RT interface during testis development.

## 1 Introduction

The rete testis (RT) is a complex network of interconnected channels and cavities that links seminiferous tubules to efferent ductules, enabling the transport of spermatozoa from the testis to the epididymis. Historically, the RT was largely regarded as serving mainly a transport function; however, recent research has revealed additional roles for the RT in shaping the regional specialization of Sertoli cells (SCs) at the terminal segments of seminiferous tubules (Imura-Kishi et al., 2021; Uchida et al., 2022). These terminal segments have been described as a niche for spermatogonial stem cells (Aiyama et al., 2015) and as a site that retains long-term proliferating SCs (Figueiredo et al., 2016; 2019). Despite these advances, the developmental origin of RT cells and their relationship to SCs remain incompletely resolved.

Testis development initiates around embryonic day 9.5 (E9.5), when cells of the coelomic epithelium covering the ventromedial surface of the mesonephros start to migrate inward, forming the bipotential gonadal primordium (Cool et al., 2012; Nef et al., 2019). These cells give rise to the SC lineage and interstitial cell populations. SC precursors cease migrating by E11.4, whereas migration of other cells continues until around E12.5 (Karl and Capel, 1998). By E10.5, expression of *Sry* and its direct downstream target *Sox9* is initiated in XY gonads, indicating the onset of sexual differentiation (Nef et al., 2019). Supporting cell precursors differentiate into SCs and assemble into testis cords (Cool et al., 2012). These cords later mature into seminiferous tubules, whereas the epididymis and efferent ductules arise from mesonephric tissue (Joseph et al., 2009).

RT morphogenesis occurs at the gonad-mesonephros interface and proceeds through multiple stages. Early SF-1-positive gonadal cells appear adjacent to mesonephric tubules as early as on E10.5, indicating the establishment of connectivity between the forming testis cords and the mesonephros (Omotehara et al., 2020). By E12.5, RT cells are predominantly localized near the mesonephric region and can be visualized by SOX9 and PAX8 immunostaining (Kulibin and Malolina, 2020). Between E13.5 and E14.5, AMH^+^ cords on the mesonephric side of the gonad remodel in a graded manner along the anteroposterior axis, and testis cord-RT connections progressively lose AMH expression. At the same time, cells with intermediate phenotypes (PAX8^+^/AMH^+^ and DMRT1^+^/AMH^−^) appear in the proximal RT region adjacent to the testis cords, suggesting potential transitions between SC and RT cells (Kulibin and Malolina, 2020).

Single-cell RNA sequencing (scRNA-seq) has become one of the key approaches for studying gonadal embryogenesis. Recent RNA-seq studies report transcriptional similarities between RT cells and SCs, including shared expression of *Nr5a1*, *Wt1*, and *Sox9* (Mayère et al., 2022; Uchida et al., 2022; Malolina et al., 2023). In this study, we use the term supporting cells to collectively refer to pre-Sertoli cells (preSC), defined here as SC precursors, SCs, and RT populations. This grouping follows the terminology used in recent single-cell studies of gonadal development (Mayère et al., 2022; Garcia-Alonso et al., 2022)**.** Lineage reconstruction analyses further support the possibility of plasticity between RT cells and SCs (Mayère et al., 2022). Notably, cell cycle profiling suggests low proliferative activity of the embryonic RT cell population (Mayère et al., 2022), indirectly supporting the idea that RT expansion may require an external cellular input.

Here, we combine chimeric organotypic culture of embryonic urogenital complexes (UGCs) with a reanalysis of a published scRNA-seq dataset (Mayère et al., 2022) to trace the origin of RT cells. Specifically, we test the hypothesis that a subset of PAX8^+^ RT cells derives from the SC lineage. Our findings refine current models of RT formation and illuminate cellular relationships at the RT cell-SC interface.

## 2 Materials and methods

### 2.1 Experimental animals

Adult male and female C57BL/6 mice (2–3 months old) were purchased from the “Stolbovaya” breeding center of the Scientific Center of Biomedical Technologies (Russia) and housed in the vivarium of the Koltzov Institute of Developmental Biology, Russian Academy of Sciences (IDB RAS). Male C57BL/6-Tg (ACTB-EGFP)1Osb/J mice with EGFP expression driven by the β-actin promoter were maintained on the C57BL/6 background. The animals were kept in standard vivarium conditions with a 12 h light/dark cycle and with food and water provided *ad libitum*.

To obtain timed pregnancies, females were housed with males at 17:00. Vaginal plugs were checked no later than at 12 h of the following day, and the day of plug detection was defined as E0.5. The embryos were collected at E12.5-E16.5.

All experimental procedures involving animals were conducted in compliance with the European Convention for the Protection of Vertebrate Animals Used for Experimental and Other Scientific Purposes and were approved by the Animal Care and Use Committee of the Koltzov Institute of Developmental Biology, Russian Academy of Sciences (Moscow, Russia).

### 2.2 Dissection of embryonic UGCs

The embryos were dissected in sterile physiological saline, and sex determination was carried out under a stereomicroscope by visual identification of the testis cords present in male gonads. UGCs, each composed of the developing testis and the adjacent mesonephros, were isolated using 25G injection needles. Fine dissection of UGCs was performed with 30G dental needles. All procedures were carried out in DMEM/F12 medium (PanEco) within a maximum of 3 h.

### 2.3 Organotypic culture

Male UGCs dissected on E12.5 were used for all organotypic culture variations. All explants were cultured under identical conditions at the liquid/gas interface on agar blocks following the protocol of Capel and Batchvarov (2008) at 37 °C with 5% CO_2_ for 72 h.

The agar blocks were prepared by boiling 1.5% Bacto-Agar (Sigma) in DMEM/F12 medium for 5 min, after which 1.5 mL of the solution was cast into a mold. After solidification, the blocks were transferred into 35-mm Petri dishes. At least 2 h prior to culture initiation, the agar blocks were saturated with 1.5 mL of DMEM/F12 culture medium supplemented with alanyl-glutamine (PanEco), sodium pyruvate (PanEco), penicillin/streptomycin (PanEco), and 10% fetal bovine serum (Gibco). Immediately before culture, the saturation medium was partially removed and 300 µL of fresh medium was added to each Petri dish. Dissected UGCs were then placed into narrow grooves molded in the agar blocks using a glass capillary. The culture medium was changed every 24 h. Each experiment was performed with at least three replicates.

### 2.4 Generation of chimeric UGCs

Chimeric UGCs were generated by combining dissected parts of UGCs from GFP-negative and GFP-positive embryos. Embryonic tissues (mesonephroi and gonads) were transferred into grooves formed in an agar block using a glass capillary. Excess fluid was carefully removed from the grooves, and embryonic tissue fragments were positioned so that their cut surfaces were in close contact to facilitate subsequent fusion (Martineau et al., 1997; Capel and Batchvarov, 2008; Imaimatsu et al., 2023). The resulting chimeric recombinants were then cultured as described above.

### 2.5 Immunofluorescence staining

The samples were fixed for 24 h in 10% buffered formalin at 4 °C, dehydrated, embedded in paraffin, and serially sectioned with a 4 µm thickness. The sections were deparaffinized and were subjected to antigen retrieval by boiling in citrate buffer (trisodium citrate dihydrate (10 mM); Tween-20 (0.05%); pH 6.0) for 50 min. Next, the sections were incubated for 30 min at 37 °C in blocking solution consisting of PBS supplemented with 3% bovine serum albumin, 0.1% Tween-20, and 0.1% Triton X-100. Subsequently, the sections were incubated with primary antibodies diluted in blocking solution without Triton X-100 either for 60 min at 37 °C or overnight at 4 °C, followed by incubation with secondary antibodies for 30 min at 37 °C.

Sequential immunohistochemical staining and antibody elution from the tissue sections were performed according to Omotehara et al. (2020). Briefly, the sections were first stained for AMH, PAX8, and NR5A1 using the immunostaining procedure described above. Antibodies were then eluted following Omotehara et al. (2020) by incubating the sections for 50 min at 60 °C in stripping buffer containing 1% β-mercaptoethanol, 2% SDS, and 62.5 mM Tris-HCl (pH 6.8). After elution, the sections were washed without shaking in distilled water and PBS, and were then reprocessed according to the same protocol for subsequent staining for SOX9.

Whole-mount immunofluorescence staining was performed by fixing samples in 4% formaldehyde in PBS for 30 min, followed by incubation in 10% methanol. The specimens were then blocked and permeabilized in PBS containing 3% BSA and 0.5% Triton X-100, and were incubated with primary antibodies for 48 h at 4 °C, followed by incubation with secondary antibodies for 48 h at 4 °C.

The primary antibodies used included antibodies to PDGFRA (rabbit monoclonal, Abcam, ab203491, 1:500), NR5A1/SF1 (mouse monoclonal, Thermo Fisher, 434200, 1:50), SOX9 (rabbit polyclonal, Merck, AB5535, 1:200), KI-67 (rat monoclonal, Thermo Fisher, 14-5698-82, 1:200), AMH (goat polyclonal, Santa Cruz, sc-6886, 1:250), GFP (chicken polyclonal, Abcam, ab13970, 1:400), and PAX8 (rabbit monoclonal, Thermo Fisher, MA5-32382, 1:200). Corresponding secondary antibodies conjugated to Alexa Fluor 488, 555, and 647 (Thermo Fisher, 1:500) were employed. Imaging was performed using a Leica Thunder microscope and a Zeiss LSM 880 Airyscan confocal microscope.

### 2.6 3D reconstruction

Three-dimensional reconstructions were generated using automated software Voloom (microDimensions GmbH, Munich, Germany) based on serial sections of the E12.5, E14.5 and E16.5 UGCs. Segmentation was guided by staining for DAPI, PAX8, KI-67, and AMH.

### 2.7 Global clustering and annotation of single-cell data

Single-cell RNA-seq count matrices for embryonic mouse gonads (E11.5, E12.5, E13.5, and E16.5) were retrieved from the NCBI Gene Expression Omnibus (accession GSE184708, Mayère et al., 2022). Analyses were performed in R 4.4.1 with Seurat v5.0.1. The cells were stratified by developmental stage (E11.5, E12.5, E13.5, and E16.5) according to the accompanying metadata file.

The stage-specific count matrices were log-normalized to 10,000 transcripts per cell with NormalizeData. Two biological replicates per stage were integrated with FindIntegrationAnchors and IntegrateData. Because empty droplets and damaged cells had been removed by the original authors, we only inspected the number of detected genes per cell (nFeature_RNA; 1000-4500 genes/cell) and mitochondrial read percentage (<1%).

Highly variable genes (method = “vst”, n = 3,000) were used for principal-component analysis (PCA). The elbow plot indicated that the first 25 principal components (PCs) captured the relevant variance. A shared-nearest-neighbor graph was constructed with FindNeighbors (k.param = 30, dims = 1–25) and clusters were identified with the Leiden algorithm (FindClusters, resolution = 0.5). Two-dimensional embeddings were generated with UMAP (RunUMAP). Clusters were annotated manually using canonical markers, and cluster-specific genes were obtained with FindMarkers and FindAllMarkers.

### 2.8 Reclustering the male supporting cell lineage

All cells initially labeled as pre-Sertoli cell (preSC), Sertoli cell (SC), or rete testis (RT) were extracted from each integrated stage object. The subset matrix was re-normalized (NormalizeData), 2000 highly variable genes were re-identified (FindVariableFeatures), and the two replicates were re-integrated (IntegrateData).

Dimensionality reduction (PCA on the 2000 highly variable genes (HVGs); 20 PCs retained) and graph-based clustering were repeated (FindNeighbors, k. param = 30; FindClusters). The resulting sub-clusters were visualized with UMAP and annotated on the basis of newly derived markers (FindAllMarkers).

### 2.9 Differential expression and gene ontology enrichment analysis

For each developmental stage (E11.5, E12.5, E13.5, and E16.5), we identified stage-specific signatures by contrasting the *Lhx9* ^+^ SC clusters and preSC-5 clusters with all other SC and pre-SC clusters at the same stage (FindMarkers, Seurat v5.0.1; |log_2_FC| > 0.25, *min. pct* ≥ 0.10, Benjamini–Hochberg FDR <0.05). Genes with positive or negative log_2_FC (Fold Change) were retained as “upregulated” and “downregulated” lists, respectively.

The eight resulting gene sets (Up and Down for each stage) were analyzed simultaneously with compareCluster (clusterProfiler v4.10), using the *enrichGO* function, the *Biological Process* ontology, the mouse annotation database org.Mm.eg.db, and the expressed gene universe of the corresponding Seurat object. Multiple testing correction employed the Benjamini-Hochberg method (adjusted *P* < 0.05). The enrichment results were visualized as dot plots that displayed the ten most significant GO terms per gene set.

### 2.10 Cell-cell communication analysis

For each embryonic stage (E11.5-E16.5), we ran CellChat (v 1.6.1) on stage-specific Seurat objects restricted to the supporting-cell clusters that included preSCs, SCs and their SC-Lhx9 sub-clusters, the C5 and the RT clusters; all other gonadal or mesonephric cell types were excluded. Clusters containing fewer than 30 cells were removed with filterCommunication (min Cells = 30), after which the standard CellChat workflow (probability inference, network construction, and pathway analysis) was executed without further modifications.

### 2.11 RNA velocity analysis

For each developmental stage (E11.5, E12.5, E13.5, and E16.5), RNA velocity was computed exclusively on the reclustered dataset comprising preSCs, SCs, and RT clusters. Spliced and unspliced counts were generated with Velocyto (v0.17.17); velocity vectors were then estimated with scVelo (v0.3.3) in stochastic mode following the official scVelo workflow.

## 3 Results

### 3.1 RT development proceeds under organotypic culture conditions

**FIGURE 1:**
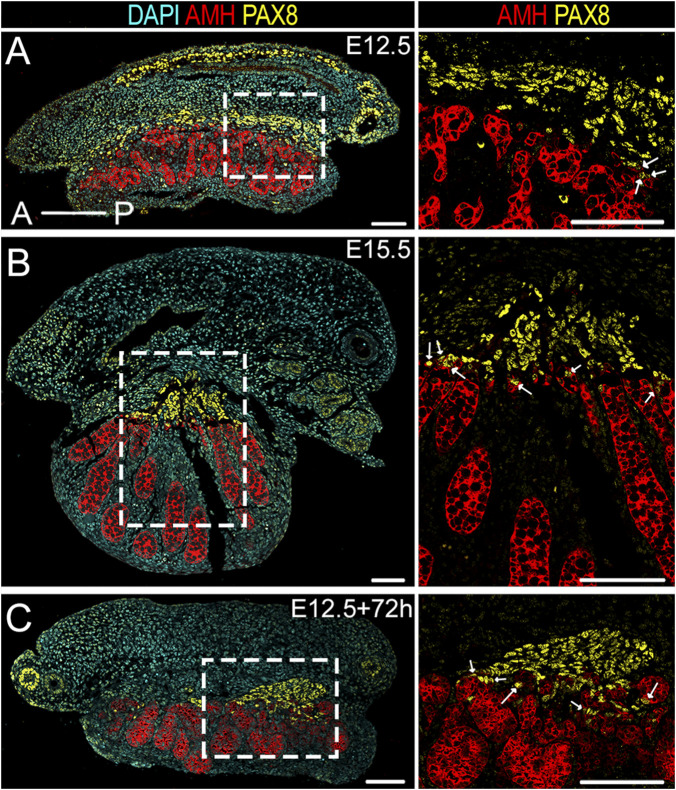
Histological structure of male UGCs. Representative images of intact E12.5 **(A)** and E15.5 **(B)** UGCs and an E12.5 UGC after 72 h of culture **(C)**, immunostained for AMH and PAX8. The panels on the right show magnified images of boxed areas; the arrows indicate PAX8^+^/AMH^+^ double-positive cells. All images are composites stitched from several adjacent fields of view. Directional axes are indicated as follows: A, anterior; P, posterior. All images were acquired by confocal microscopy. Scale bars: 100 µm.

By E12.5, RT cells formed a network of cords along the anterior-posterior axis of the male urogenital complex (UGC), which in this study denotes the mesonephros-gonad complex. Most RT cells resided in the anterior third, where the network contacted mesonephric tubules on one side (distal region of the RT) and testis cords on the other (proximal region of the RT). AMH was used as a marker of SCs, whereas PAX8 labeled the RT. A few transitional PAX8^+^/AMH^+^ cells were present at the border between the RT and testis cords (Figure 1A). Under normal development on E15.5, the RT occupied a central position within the UGC and extended protrusions that contacted all testis cords. Transitional PAX8^+^/AMH^+^ cells were located at these interfaces (Figure 1B). After 72 h of organotypic culture of E12.5 male UGC explants, RT cells remained predominantly in the anterior third of the gonad, which exhibited a more elongated shape compared with *in vivo* E15.5 testes. Numerous PAX8^+^/AMH^+^ cells were observed within protrusions interfacing with the testis cords (Figure 1C). These findings indicate that RT development, in particular, the emergence of PAX8^+^/AMH^+^ cells, is preserved under these *in vitro* conditions, although its spatial positioning remains anteriorized and developmental progression is delayed relative to intact testes. Accordingly, this system represents a valuable tool for studying the mechanisms underlying RT formation.

### 3.2 The topography of the RT in the UGC and the methodological design for gonadal dissection

**FIGURE 2:**
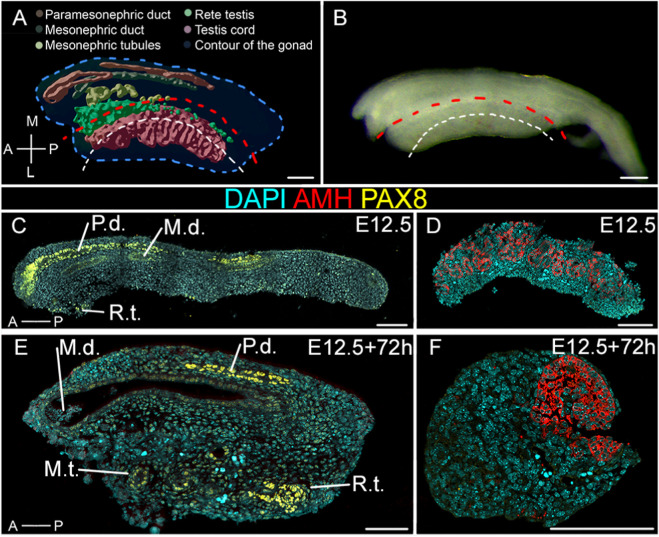
Topography of the RT and a dissection scheme of the E12.5 male UGC **(A)** 3D reconstruction of the E12.5 UGC was generated from serial histological sections immunostained for AMH and PAX8 **(B)** A representative image of an intact E12.5 male UGC. Dissection incisions are indicated by red dashed lines **(A,B)** for isolating the mesonephros devoid of SCs and by white dashed lines for isolating the testis fragment free of RT cells **(C,D)** Representative sections of E12.5 mesonephric **(C)** and testis **(D)** fragments after dissection, stained for AMH and PAX8 **(E,F)** Representative sections of E12.5 mesonephric **(E)** and testis **(F)** fragments after 72 h of organotypic culture stained for AMH and PAX8. Directional axes are indicated as follows: A, anterior; P, posterior; M, medial; L, lateral. P.d., paramesonephric duct; M.d., mesonephric duct; M.t., mesonephric tubule; RT, rete testis. **(C,D)** are composites stitched from several adjacent fields of view; images in **(C–F)** were acquired by confocal microscopy. Scale bars: 100 µm.

Prior to initiating tissue-recombination organ culture assays to investigate RT ontogeny, we optimized the dissection protocol described by (Capel and Batchvarov, 2008) for E12.5 mouse UGCs. To delineate the topography of the RT on E12.5, we generated a series of paraffin sections of a mouse male UGC immunostained for PAX8 and AMH. From these sections, we reconstructed a high-resolution 3D model mapping the precise localization of AMH^+^ SCs and PAX8^+^ RT cells. The mesonephric duct, the paramesonephric duct, and mesonephric tubules whose cells also expressed PAX8 were distinguished from the RT by the presence of a luminal space and their characteristic anatomical positions (Figure 2A).

Analysis of the model allowed us to define the lower boundary reached by RT cells and to determine the lines of dissection incisions for isolating pure mesonephric fragments and gonadal fragments (Figures 2A,B). To isolate a pure mesonephric fragment devoid of SCs, an UGC was transected precisely along the gonad-mesonephros interface and higher RT/testis cords boundary (red dashed lines in Figures 2A,B). The purity of the isolated mesonephroi was confirmed by AMH immunostaining: all six dissected samples (6/6) lacked AMH^+^ cells, whereas a small portion of the distal RT was retained (Figure 2C). To obtain testis tissue free of RT cells, we made an incision approximately one-third of the gonad’s thickness from the gonad-mesonephros boundary toward the gonad (white dashed lines in Figures 2A,B). The purity of these gonadal preparations was assessed by PAX8 immunostaining: in eight out of eight samples (8/8), no PAX8^+^ cells were detected (Figure 2D).

After refining the UGC dissection protocol, we examined whether PAX8^+^ cells would appear in E12.5 testis explants and whether AMH^+^ cells would appear in E12.5 mesonephros explants after 72 h of culture. In mesonephric organotypic cultures, none of the six replicates (6/6) contained AMH^+^ cells (Figure 2E). In gonadal organotypic cultures, seven out of eight (7/8) samples lacked PAX8^+^ cells (Figure 2F). Only one replicate contained both PAX8^+^/AMH^+^ and PAX8^+^/AMH^−^ cells likely reflecting a minor dissection error that inadvertently included RT cells.

### 3.3 PAX8^+^ cells emerge in the gonadal portion of the chimeric male UGC during organotypic culture

**FIGURE 3:**
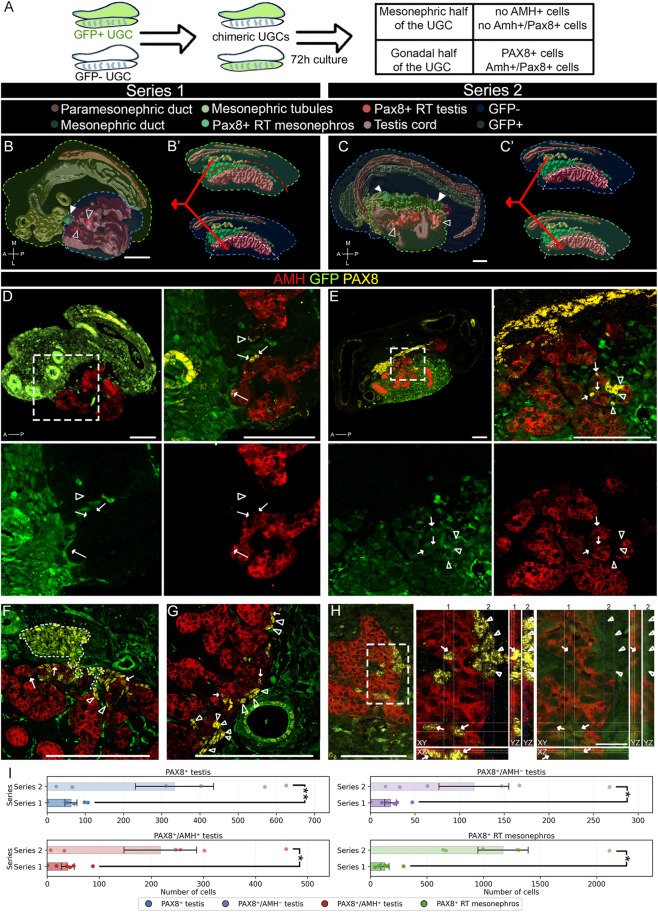
Emergence of PAX8^+^ cells in chimeric male E12.5 UGCs after 72 h of culture. **(A)** Scheme of the experiment. **(B–H)** Representative images of results obtained in the first **(B,D)** and second **(C–H)** experimental series of chimeric organotypic cultures **(B,C)** 3D reconstructions of representative samples from experimental series 1 **(B)** and series 2 **(C,B′,C′)** schemes illustrating the assembly of chimeric UGCs. Hollow arrowheads mark PAX8^+^ cells derived within the gonadal compartment of the UGC; arrowheads mark PAX8^+^ cells derived within the mesonephric compartment of the UGC. **(D,E)** Representative images of the sections from **(B,C)** stained for AMH, PAX8, and GFP. The adjacent panels show magnified images of boxed areas from **(D,E)**. **(F–H)** Additional images of chimeric UGCs from experimental series 2, **(F)** illustrating the connection of testis-derived PAX8^+^ structures with the RT from the mesonephric compartment (outlined by a dashed line), **(G)** the position of these structures along the gonad-mesonephros border, and **(H)** and testis-derived PAX8^+^/AMH^+^ cells within testis cords. The adjacent panels show magnified views of the boxed region in **(H)**; lines mark the boundaries of the XY and YZ orthogonal projections. On **(F,G)** GFP^+^ mesonephros/GFP^−^ gonad **(H)** GFP^−^ mesonephros/GFP^+^ gonad chimeric organotypic cultures. In D-H, arrows indicate double-labeled PAX8^+^/AMH^+^ cells from the gonadal compartment of the UGCs, and hollow arrowheads mark PAX8^+^/AMH^−^ cells from the gonadal compartment. Panels D–H were acquired using confocal microscopy; panels D–E show composites stitched from multiple adjacent fields of view. **(I)** Quantification of PAX8^+^ cells in the gonadal region of the UGC (PAX8^+^ testis) and in the distal RT of the mesonephric portion of the UGC (PAX8^+^ mesonephros) from the first and second experimental series. The data are presented as the number of cells per sample (mean ± SEM; n = 6). *p ≤ 0.05; **p ≤ 0.01 (one-way ANOVA). Directional axes are indicated as follows: A, anterior; P, posterior; M, medial; L, lateral. Scale bars. **(B–G)** 200 μm, **(H)** 100 μm; magnified images 20 µm.

To generate chimeric male UGC organotypic cultures, we used male UGCs obtained from C57BL/6 (GFP^−^) embryos and from embryos carrying an ACTB-EGFP transgene (GFP^+^), and combined tissues from these two donor types in culture. Chimeric cultures were generated following the approach described by (Capel and Batchvarov, 2008). Chimeric male UGCs (GFP^+^ mesonephros/GFP^−^ gonad or GFP^−^ mesonephros/GFP^+^ gonad; Figure 3A) were cultured for 72 h, after which the emergence of PAX8^+^ cells in the gonadal regions of the UGCs was assessed. We examined the presence of PAX8^+^/GFP^+^ cells when the gonadal portions of chimeric UGCs were from GFP^+^ embryos and the presence of PAX8^+^/GFP^−^ cells when the gonadal portions were from GFP^−^ embryos. In five out of six (5/6) chimeric UGCs, PAX8^+^ cells emerged in the gonadal portion, some of them co-expressed AMH (Figures 3B,B’,D). PAX8^+^/AMH^+^ cells were localized within testis cords, whereas PAX8^+^/AMH^−^ cells were detected outside the cords (Figure 3D). We also examined the emergence of AMH^+^ cells in the mesonephric regions of chimeric UGCs, specifically AMH^+^/GFP^+^ cells when the mesonephric portions of chimeric UGCs were from GFP^+^ embryos and AMH^+^/GFP^−^ cells when the mesonephric portions were from GFP^−^ embryos. No AMH^+^ cells were identified in the mesonephric regions (Figure 3D). In addition, we observed GFP-labeled mesonephric-derived cells within the gonadal compartment (Figures 3D,F), which is consistent with the male-specific mesonephric cell migration into the developing gonad described by Martineau et al. (1997).

As PAX8^+^ cells did not emerge in cultured testis explants devoid of a mesonephros region with distal RT (Figure 2F), we hypothesized that the distal RT might induce *Pax8* expression in testicular cells. To test this, we performed the second experimental series where we aimed to preserve as much of the distal RT as possible within the mesonephric portion, allowing the inclusion of some SCs. For this purpose, UGCs were dissected slightly below the gonad-mesonephros boundary (between the white and red dashed lines in Figure 2B). The gonadal portion was obtained as described above. In all nine chimeric UGCs (n = 9), PAX8^+^ cells emerged in the gonadal compartment. Similarly to series 1, PAX8^+^/AMH^+^ cells were localized within testis cords, whereas PAX8^+^/AMH^−^ cells were detected outside the cords, immediately adjacent to their periphery (Figures 3C,C’,E).

In additional representative samples from experimental series 2, PAX8^+^ cells arising within the gonadal compartment formed continuous structures connected with the RT of the mesonephric compartment (Figure 3F). These testis-derived PAX8^+^ structures could be extensive and extended along the anterior–posterior axis of the UGC at the testis–mesonephros border mirroring normal embryonic RT anatomy (Figure 3G). A subset of the newly formed PAX8^+^ cells was localized among SCs within testis cords and co-expressed AMH as shown in the orthogonal projections of serial confocal optical sections (Figure 3H).

Complete serial sections suitable for cell counting were obtained for chimeric UGCs of the two series (6 samples per series), and only these samples were included in the quantitative analysis. The remaining three samples of series 2 were assessed only qualitatively. Quantitative analysis revealed that a higher number of PAX8^+^ cells in the distal RT of the chimeric UGC mesonephric region (1,171.8 ± 222.5 cells per UGC in the second series vs. 129 ± 44.6 cells in the first series, p = 0.001) significantly increased the number of newly formed PAX8^+^ cells in the gonadal region of UGCs (333.3 ± 102.3 cells vs. 62.5 ± 15.9 cells respectively, p = 0.026; Figure 3I). In addition, within the gonadal compartment, we observed increases in both PAX8^+^/AMH^−^ (116.0 ± 39.2 cells vs. 22.7 ± 6.5 cells, p = 0.041) and PAX8^+^/AMH^+^ (217.3 ± 69.8 cells vs. 39.8 ± 12.3 cells, p = 0.031) subsets in the second series compared with the first series (Figure 3I). The proportion of PAX8^+^/AMH^+^ cells among all PAX8^+^ cells in the gonadal region was comparable between the two series and was 0.62 ± 0.08 in series 1 and 0.58 ± 0.07 in series 2.

A methodological limitation of chimeric UGC-based lineage contribution analyses is their sensitivity to dissection precision: inadvertent inclusion of adjacent structures can introduce artefacts (e.g., transfer of pre-existing mesonephric PAX8^+^ cells into the gonad). Although the dissection protocol was carefully calibrated, PAX8^+^ cells were detected in one out of eight cultured testis controls (see the Results section above), most plausibly reflecting a rare dissection error. In contrast, PAX8^+^ cells were detected within the testis in 14 out of 15 experimental chimeric UGCs in total. These proportions differed significantly between groups (Fisher’s exact test, p = 0.00025), making a dissection artefact explanation for PAX8 emergence in the gonadal compartment highly unlikely.

### 3.4 The heterogeneity of the testicular supporting cell lineage based on single-cell RNA sequencing data analysis

**FIGURE 4:**
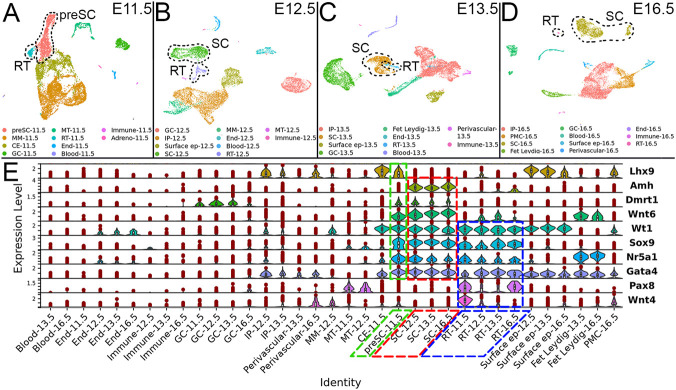
Bioinformatics analysis of single-cell RNA-sequencing data from male mouse embryonic UGCs at E11.5 and testes at E12.5, E13.5, and E16.5. **(A–D)** UMAP projections of cell clusters at each developmental stage. Colors indicate technical cluster identities and are used only to distinguish clusters visually; cluster names were assigned by manual annotation based on canonical marker genes. Two biological replicates were analyzed for each developmental stage. **(E)** A violin plot showing the expression of key marker genes for each annotated cell population. Colors indicate individual genes for visual distinction. Dashed outlines indicate marker genes. Data were log-normalized to 10,000 transcripts per cell; highly variable genes were used for PCA, graph-based Leiden clustering, UMAP visualization, and marker-based cluster annotation. Adreno, adrenal cells; CE, coelomic epithelium; End, endothelial cells; Fet Leydig, fetal Leydig cells; GC, germ cells; Immune, immune cells; IP, interstitial progenitors; MM, mesonephric mesenchyme; MT, mesonephric tubules; Perivascular, perivascular cells; PMC, peritubular myoid cells; preSC, Sertoli-cell precursors; RT, rete testis cells; SC, Sertoli cells; Surface ep, surface epithelium.

We reanalyzed the single-cell RNA sequencing dataset of embryonic mouse testes obtained by Mayère et al. (GSE184708, originally published in Mayère et al., 2022) to identify rare or transitional cell populations potentially involved in RT formation. Using this resource, we profiled 10,398 cells at E11.5, 10,602 cells at E12.5, 10,602 cells at E13.5, and 9,209 cells at E16.5. To maximize the accuracy of cell-population identification, unsupervised clustering was performed independently for each developmental stage (Figures 4A–D). Differential gene expression analysis was then conducted to define cluster-specific marker genes (Supplementary Material 1).

Clusters were annotated using known markers of embryonic testicular cell types reported previously (Mayère et al., 2022; Garcia-Alonso et al., 2022). Specifically, Sertoli cells (SCs) were characterized by a high expression of *Amh, Sox9*, *Gata4, Nr5a1, Wnt6*, *Wt1*, and *Dmrt1*, while their progenitors (preSCs) expressed the same genes except *Amh* and also exhibited a high expression of *Lhx9*. RT cells expressed *Sox9*, *Nr5a1, Wt1*, *Gata4*, and *Pax8. Wnt4* was identified as an RT cell marker at early developmental stages, however, *Wnt4* transcripts were not detected in RT cells by E16.5 (Figure 4E). We identified 1784 preSCs and 254 RT cells at E11.5, 1809 SCs and 299 RT cells at E12.5, 2134 SCs and 198 RT cells at E13.5, and 2346 SCs and 45 RT cells at E16.5.

Interestingly, we observed a marked heterogeneity in the degree of *Pax8* expression within the RT cluster across E11.5-E16.5*.* In addition, RT cells at some developmental stages expressed preSC marker *Lhx9* and SC markers *Wnt6* and *Amh* (Figure 4E).

**FIGURE 5:**
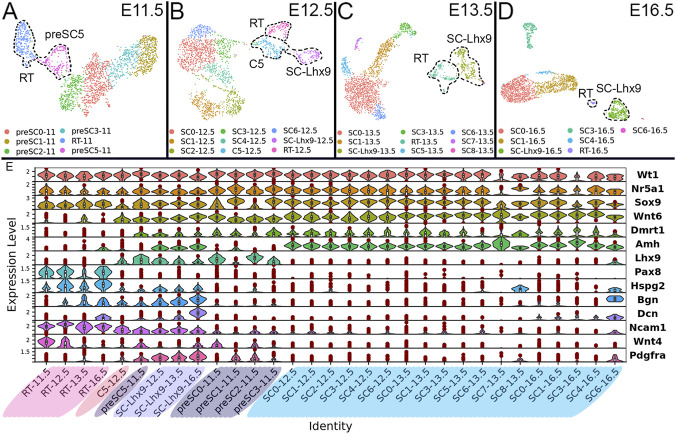
Reclustering preSCs, SCs, and RT cells at E11.5, E12.5, E13.5, and E16.5. **(A–D)** UMAP projections of cell clusters at each developmental stage. Colors indicate technical subcluster identities and are used only to distinguish subclusters visually; subcluster names were assigned by manual annotation based on marker-gene expression. Two biological replicates were analyzed for each developmental stage. **(E)** A violin plot showing expression levels of key marker genes. Colors indicate individual genes for visual distinction. Reclustering was performed after extraction of preSC, SC, and RT cells from the stage-specific integrated objects, followed by re-normalization, selection of highly variable genes, PCA, graph-based clustering, UMAP visualization, and marker-based annotation. preSC, Sertoli-cell precursors; RT, rete testis cells; SC, Sertoli cells.

To investigate the heterogeneity within the testicular supporting cell lineage more thoroughly, we performed a focused reclustering limited to these populations. At E11.5, five preSC sub-clusters (n = 1754) and one RT cluster (n = 284) were identified (Figures 5A,E). At E12.5, seven SC sub-clusters (n = 1807), one RT cluster (n = 149), and an additional cluster C5 (n = 152) were identified (Figures 5B,E). Cluster C5 co-expressed genes common to both SC and RT cells, including *Wt1*, *Nr5a1*, and *Sox9*, and showed expression of SC marker *Wnt6* (Garcia-Alonso et al., 2022), while *Amh* was absent or only weakly detected. In addition, this cluster also expressed the undifferentiated gonadal somatic cell marker *Lhx9* (Garcia-Alonso et al., 2022) and RT-enriched genes, including *Ncam1* (Møller et al., 1991) and low levels of *Wnt4* (Mayère et al., 2022). Notably, *Pax8* transcripts were not detected in this population. Cells from C5 also expressed proteoglycans perlecan (*Hspg2)* and (*Bgn)*, which are characteristic of RT cells, according to our RT marker analysis (Figure 5E).

By E13.5, the dataset comprised one RT cluster (n = 162) together with eight SC sub-clusters (n = 2,170) (Figures 5C,E). At E16.5, one RT cluster (n = 42) and six SC sub-clusters (n = 2,349) were detected (Figures 5D,E). The changes in the numbers of cells within the SC and RT clusters, as well as the appearance of cluster C5 at E12.5, reflect the higher resolution enabled by the focused reclustering procedure.

For stages E12.5, E13.5, and E16.5, we resolved sub-clusters SC-Lhx9-12.5, SC-Lhx9-13.5, and SC-Lhx9-16.5 (136, 279, and 401 cells respectively) that share highly similar transcriptional profiles. All three sub-clusters co-expressed canonical SC markers (*Amh, Dmrt1,* and *Wnt6*) together with *Lhx9* and *Pdgfra* expressed by preSCs and cells from the C5 cluster. Strikingly, they also exhibited expression of RT markers *Ncam1* and proteoglycans decorin (*Dcn*), *Bgn*, and *Hspg2. Wnt4* showed low expression at E12.5 and E13.5, whereas its expression increased by E16.5 (Figure 5E).

At E11.5, the preSC5-11.5 (176 cells) cluster exhibited an expression pattern closely matching that of C5-12.5 and SC-Lhx9-12.5, characterized by the expression of *Ncam1, Dcn, Bgn*, and *Hspg2* (Figure 5E).

### 3.5 RNA velocity-based modeling of differentiation trajectories of the testicular supporting cell lineage

RNA velocity is a computational method that uses the relative levels of unspliced versus spliced RNA to estimate, for each cell, the direction of ongoing gene expression change, indicating where the cell is headed next in a transcriptional space. When projected onto a UMAP embedding, these velocity vectors help model the direction of cell differentiation by suggesting probable transitions between cell states along a developmental trajectory (La Manno et al., 2018).

**FIGURE 6:**
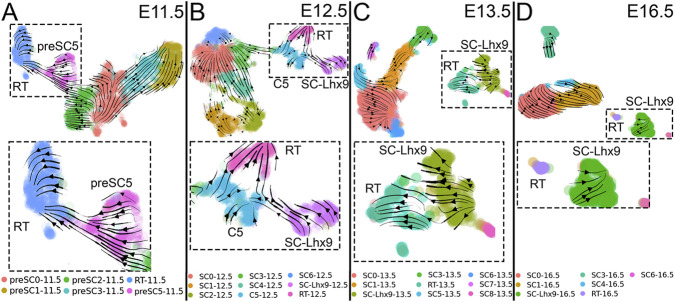
RNA velocity analysis preSCs, SCs, and RT cells. RNA velocity trajectory maps overlaid on UMAPs for the corresponding stages, specifically, E11.5 **(A)**, E12.5 **(B)**, E13.5 **(C)**, and E16.5 **(D)**. The arrows indicate RNA velocity vectors. The bottom panels show magnified images of boxed areas from **(A–D)**.

We inferred RNA velocities using Velocyto (v0.17.17) to quantify spliced and unspliced reads and scVelo (v0.3.3) to estimate velocities, using the published scRNA-seq dataset from Mayère et al. (2022). Velocity streamlines projected onto the UMAP embedding revealed a clear transcriptional flow (Figure 6). On E11.5, streamlines originated in the preSC5-11.5 sub-cluster and converged on RT-11.5, suggesting a preSC to RT trajectory (Figure 6A). On E12.5, trajectories ran from the SC-Lhx9-12.5 and the C5-12.5 sub-clusters towards RT-12.5, with C5 also showing trajectories towards SC-Lhx9 (Figure 6B). On E13.5, streamlines originated from SC-Lhx9-13.5 and terminated in the RT-13.5 sub-cluster (Figure 6C). By E16.5, velocity vectors converging on RT were absent (Figure 6D). These *in silico* trajectories suggest that preSCs, C5, and SCs may give rise to RT cells on E11.5-E13.5.

### 3.6 Immunohistochemical identification of the SC-lhx9-12.5 and C5 clusters in the male UGC

**FIGURE 7:**
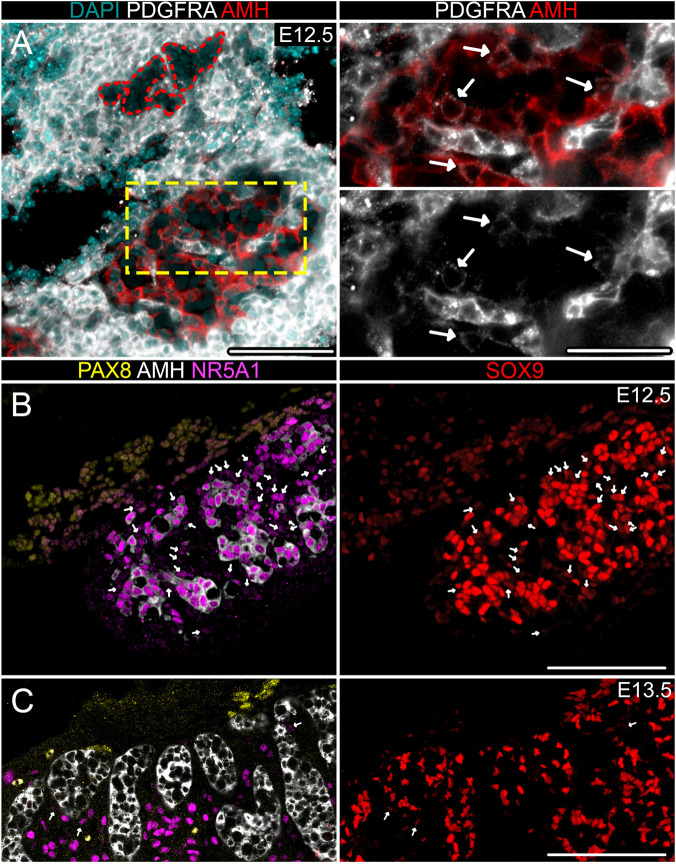
Immunohistochemical localization of cells from the SC-Lhx9-12.5 and C5 clusters in male UGCs. **(A)** A representative optical section of an E12.5 UGC whole-mount sample stained for AMH and PDGFRA. A red dashed line outlines the RT defined as the PDGFRA^−^/AMH^−^ area. The panels on the right show magnified images of the boxed area; the arrows mark AMH^+^/PDGFRA^+^ cells among AMH^+^ SCs **(B,C)** A representative image of a **(B)** E12.5 UGC and **(C)** E13.5 UGC sequentially stained for PAX8, AMH, NR5A1, and SOX9. The arrows mark PAX8^-^/AMH^−^/NR5A1^+^/SOX9^+^ cells. The panels are composites stitched from several adjacent fields of view. The images were acquired by confocal microscopy. Scale bars **(A)** 50 μm **(B,C)** 100 μm, magnified images 25 µm.

Our bioinformatics analysis revealed that the platelet-derived growth factor receptor *Pdgfra*, a marker of many testicular interstitial cells and mesonephric mesenchymal cells (Brennan et al., 2003), was also present in SC sub-clusters SC-Lhx9-12.5, SC-Lhx9-13.5, and SC-Lhx9-16.5 (Figure 5E). Guided by this expression signature, we performed PDGFRA immunohistochemistry and detected a strong membrane-associated PDGFRA signal in the testicular interstitium and in the mesonephros, together with a weak PDGFRA signal in AMH^+^ SCs located near the RT on E12.5 (Figure 7A), thereby likely localizing the SC-Lhx9-12.5 cluster *in situ*. Notably, RT cells themselves were PDGFRA^−^/AMH^−^.

The C5 cluster was defined by co-expression of *Sox9* and *Nr5a1* with no expression of *Pax8* or *Amh* (Figure 5E). To visualize this population, we applied sequential immunohistochemistry with an initial round of staining for PAX8, NR5A1, and AMH followed by antibody stripping according to Omotehara et al. (2020), while in the second round, staining for SOX9 was performed. This approach uncovered PAX8^-^/AMH^−^/NR5A1^+^/SOX9^+^ cells in E12.5 and E13.5 testes, which were likely from the C5 cluster (Figures 7B,C). These cells were localized in the gonadal interstitium between testis cords, and some of them were detected in the vicinity of the RT. Notably, by E13.5 their abundance visually appeared markedly reduced compared with E12.5.

### 3.7 Contribution of RT proliferation to RT expansion

We analyzed the distribution of cell cycle phases within the SC, C5, SC-Lhx9, and RT clusters on E12.5, E13.5, and E16.5. For this purpose, cell cycle phase scores from the dataset of Mayère et al. (2022) were reassigned to the clusters obtained in our focused analysis (Figure 5).

**FIGURE 8:**
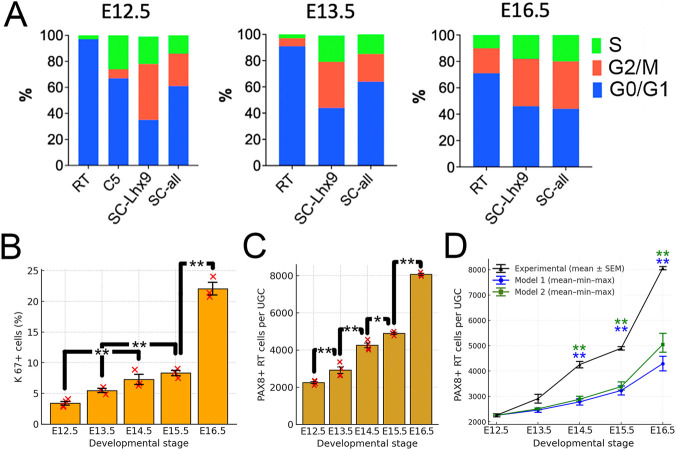
Proliferation and dynamics of RT cell numbers during embryonic stages E12.5–E16.5. **(A)** The proportions of RT, C5, SC-Lhx9, and all other Sertoli cell clusters (SC-all) in the S, G2/M, and G0/G1 phases, as inferred from *in silico* cell cycle analysis at E12.5, E13.5, and E16.5, based on our reanalysis of the single-cell RNA-seq dataset of Mayère et al. (2022). The S phase is shown in green, G2/M in red, and G0/G1 in blue. **(B)** The percentage of KI-67^+^ RT cells from E12.5 to E16.5; data are represented as mean ± SEM; at least three samples were analyzed per stage. **(C)** The numbers of PAX8^+^ RT cells per UGC from E12.5 to E16.5; data are represented as mean ± SEM; at least three samples were analyzed per stage. **(D)** A comparison of empirical RT cell numbers from **(C)** with model-based expectations. The black line shows the experimental data, and the green and blue lines indicate the model-based expectations: Model 1 (blue, circles) uses the proliferation rate defined as the arithmetic mean of the rates measured at the start and the end of each 24-h interval, whereas Model 2 (green, squares) applies the rate from the end of the interval. **(B–D)** The red crosses denote individual samples. *p ≤ 0.05; **p ≤ 0.01. One-way ANOVA with Tukey’s *post hoc* multiple comparisons correction. **(D)** At each stage, model calculations are presented in a mean–min–max format based on the empirical KI-67 data at that stage and were analyzed using one-way ANOVA. *p ≤ 0.05; **p ≤ 0.01.

On E12.5, the proportion of actively dividing RT cells (in the S or G2/M phase) was only 2.6% (Figure 8A). This fraction increased to 9.3% on E13.5% and to 28.6% on E16.5. Cluster C5 exhibited moderate proliferative activity on E12.5, with 32.6% of cells classified as actively dividing. The SC-Lhx9 sub-cluster showed the highest proportion of actively dividing cells on E12.5 (64.7%), which declined on E13.5 (55.9%) and remained comparably high on E16.5 (53.6%). In contrast, SC-all (all other SC clusters combined) displayed an intermediate level of actively dividing cells on E12.5 (38.8%), which decreased slightly on E13.5 (36.1%) and then increased markedly by E16.5 (56.4%) (Figure 8A). Overall, SC-Lhx9 proliferated more actively than SC-all at the earlier stages (E12.5-E13.5).

Next, we analyzed the dynamics of RT cell proliferation and absolute counts of RT cells on serial sections of male UGCs stained for KI-67 and PAX8. From E12.5 to E15.5, the proliferation rate of RT cells increased only slightly with 3.4% ± 0.3% of KI-67^+^ RT cells on E12.5, 5.5% ± 0.4% on E13.5, 7.3% ± 0.8% on E14.5, and 8.3% ± 0.4% on E15.5; this rate then rose sharply to 22.1% ± 1.0% on E16.5 (*p* = 6.75 × 10^−8^) (Figure 8B). Absolute counts of PAX8^+^ RT cells increased from 2,250 ± 60 cells per UGC on E12.5 to 2,911 ± 173 on E13.5 (*p* = 0.007), 4,251 ± 127 on E14.5 (*p* = 9.33 × 10^−6^), and 4,893 ± 66 on E15.5 (*p* = 0.016), ultimately rising almost twofold to 8,059 ± 62 cells per UGC on E16.5 (*p* = 1.83 × 10^−9^) (Figure 8C).

Since the proliferation rate of RT cells remained low throughout most of the examined period, we sought to determine whether the proliferative activity of RT cells alone could account for the observed increase in their absolute number.

No direct *in vivo* measurements of the cell cycle length for RT or SC cells of mouse embryos on E12.5-E14.5 have been found. We therefore used a deliberately permissive lower-bound estimate of 12.1 h, corresponding to the cell-cycle length reported for rapidly proliferating embryonic cortical progenitors of the cerebral cortex at E12.5 (Mi et al., 2018). Because longer cell-cycle estimates would only decrease the predicted contribution of local proliferation, this value provides the most conservative test of whether proliferation alone could account for RT expansion.

We modeled the growth of PAX8^+^ RT cells in the UGC from E12.5 to E16.5. Let t_0_, … ,t_4_ denote the time points corresponding to E12.5, E13.5, E14.5, E15.5 and E16.5 respectively.

Nᵢ is the number of PAX8^+^ RT cells in the UGC at stage tᵢ. We initialized N_0_ to the empirical mean cell count on E12.5 = 2,250.

fᵢ denotes the proportion of KI-67^+^ cells at stage tᵢ.

We assumed the lower bound of the cell cycle length to be 12 h, or two cell cycles per day (n = 2).

In one cell cycle, a fraction *f* of the cells divides, so the total population is multiplied by a factor of approximately 1 + *f*. Consequently, after *n* cycles the expected fold change is (1 + *f*)^n^.

To obtain an upper estimate of the increase in the PAX8^+^ cell population, we excluded cell death from the model and assumed that all cells undergoing proliferation during the day complete their divisions.

Given these definitions, we considered two simple growth models:

#### 3.7.1 Model 1 (average interval proliferation rate)

In the first model, the proliferation rate is taken as an arithmetic mean of the rates measured at the start and the end of the interval. Mathematically, for i = 0, 1, 2, 3, we have the following formula:

$$\bar{f_{i}}=\frac{f_{i}+f_{i+1}}{2}$$

$$N_{i+1}=N_{i}{\left(1+\bar{f_{i}}\right)}^{n},n=2$$

#### 3.7.2 Model 2 (final interval proliferation rate)

In the second model, each interval uses the proliferation rate at the end of the interval. For i = 0, 1, 2, 3, this is written as follows:

$$N_{i+1}=N_{i}{\left(1+f_{i+1}\right)}^{n},n=2$$

Figure 8D summarizes the RT cell counts predicted by both growth models at each developmental stage and compares them with the empirically measured RT cell numbers. These data demonstrate that intrinsic RT proliferation is insufficient to account for the observed numerical expansion of RT cells on E14.5, E15.5, and E16.5: for both growth models, the predicted RT cell counts remain significantly lower than the empirical values (*p* ≤ 0.01) (Figure 8D).

### 3.8 RT cells proliferate preferentially in the proximal domain

**FIGURE 9:**
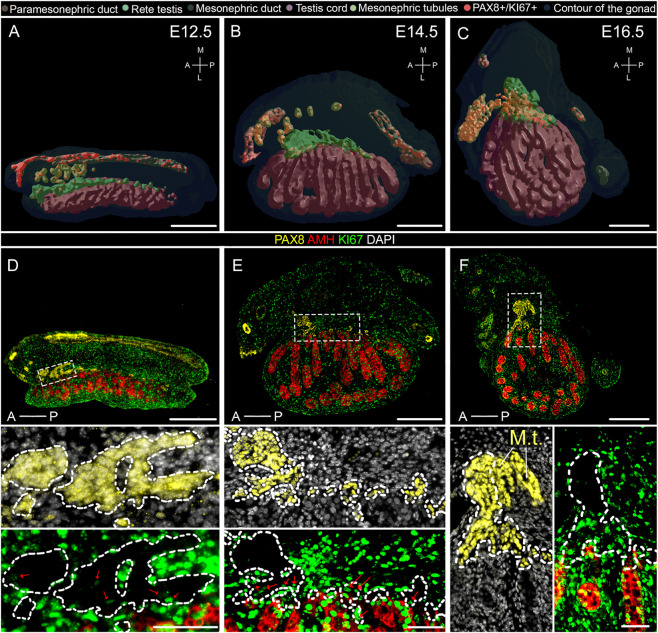
Distribution of PAX8^+^/KI-67^+^ cells in the RT at E12.5, E14.5, and E16.5. **(A–C)** 3D reconstructions illustrate the spatial distribution of PAX8^+^/KI-67^+^ proliferating cells within the RT network at E12.5 **(A)**, E14.5 **(B)**, and E16.5 **(C)**. **(D–F)** Representative images of the sections from **(A–C)** at E12.5 **(D)**, E14.5 **(E)**, and E16.5 **(F)**, stained for PAX8, KI-67, and AMH. The bottom panels show magnified images of the boxed areas from **(D–F)**; arrows indicate PAX8^+^/KI-67^+^ RT cells, and the dashed outline delineates the RT. The panels are composites stitched from several adjacent fields of view. Directional axes are indicated as follows: A, anterior; P, posterior; M, medial; L, lateral; M.t., mesonephric tubule. Scale bars **(A–F)**, 200 μm; magnified images from **(D–F)**, 100 µm.

We mapped the spatial distribution of proliferating RT cells on E12.5, E14.5, and E16.5, generating 3D reconstructions to visualize stage-specific changes (Figures 9A–C). On E12.5, PAX8^+^/KI-67^+^ cells were evenly dispersed throughout the entire RT network (Figures 9A,D). By E14.5, proliferating RT cells became mostly restricted to the proximal region, specifically, to the area directly adjacent to testis cords, while they were virtually absent from the distal RT (Figures 9B,E). A similar pattern was observed on E13.5 and E15.5 (Supplementary Figure S1). On E16.5, PAX8^+^/KI-67^+^ cells were more abundant and increasingly occupied the proximal half of the RT, whereas the distal RT, including its terminal extremity adjacent to the mesonephric tubules, remained largely devoid of these cells (Figures 9C,F).

Notably, AMH^+^/PAX8^+^ cells were localized in the same area of the RT where the proliferative activity was predominantly observed, specifically, at the RT-testis cord boundary (Figures 1A–C). Therefore, we quantified both the abundance and the proliferative status of AMH^+^/PAX8^+^ cells on E14.5. Although AMH^+^/PAX8^+^ cells constituted only (5.93% ± 0.27%) of the RT cell population at this stage, they displayed a markedly higher percentage of KI-67^+^ cells than the overall RT population (29.82% ± 2.36% vs. 4.54% ± 0.05%; one-way ANOVA, *p* = 5.19 × 10^−5^).

### 3.9 GO enrichment analysis of RT-associated supporting lineage sub-clusters in the embryonic testis

**FIGURE 10:**
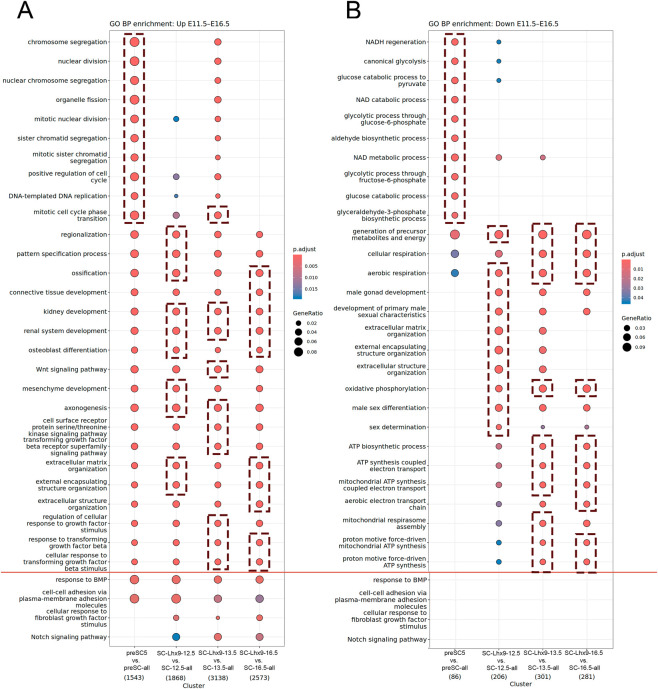
Gene Ontology over-representation analysis of DEGs in the preSC5-11.5 and SC-Lhx9 sub-clusters. For each comparison, a plot shows the top 10 enriched GO terms among the upregulated **(A)** and downregulated **(B)** gene sets, outlined with a dashed box. The dot color encodes Benjamini–Hochberg adjusted P-values, and the dot area represents the gene ratio (the number of genes associated with a given term divided by the total number of DEGs). The GO terms shown below the red line are additional illustrative terms that were not included in the top 10 for that comparison.

During the analysis of the sequencing dataset, we identified two supporting lineage subpopulations: preSC5 on E11.5 and SC-Lhx9 on E12.5-E16.5 with distinctive transcriptional features. Notably, SC-Lhx9 cells retained the molecular characteristics of early gonadal somatic lineages and additionally expressed a set of extracellular matrix (ECM) genes typically associated with the RT. The preSC5 population exhibited a transcriptional profile broadly similar to SC-Lhx9. RNA velocity further indicated that these clusters may give rise to RT cells on E11.5-E13.5. To characterize systematically which biological programs distinguish these RT-associated SC lineage clusters from the broader SC lineage, we performed GO over-representation analysis (GO-ORA) of differentially expressed genes (DEGs) based on four pairwise comparisons: preSC5-11.5 vs. preSC-all, SC-Lhx9-12.5 vs. SC-12.5-all, SC-Lhx9-13.5 vs. SC-13.5-all, and SC-Lhx9-16.5 vs. SC-16.5-all (Figure 10; Supplementary Material 2).

For preSC5 on E11.5, the top 10 upregulated GO terms were almost entirely related to the cell cycle and proliferation, including “chromosome segregation,” “nuclear division,” “organelle fission,” “sister chromatid segregation,” “DNA-templated DNA replication,” “mitotic cell cycle phase transition,” and “positive regulation of the cell cycle.” In contrast, across the SC-Lhx9 sub-clusters (E12.5-E16.5), the top 10 list was dominated by terms reflecting embryonic development, extracellular matrix organization, and signaling. Thus, on E12.5, enrichment of “pattern specification process” and “regionalization” points to tissue patterning and spatial specification programs, which constitute core components of organogenesis. Enrichment of “mesenchyme development” is consistent with mesenchymal-to-epithelial transition-related features in SC lineage cells. On E13.5, the SC-Lhx9 enrichment profile shifted toward signaling: the top 10 included “Wnt signaling pathway” and TGFβ-related categories: “transforming growth factor beta receptor superfamily signaling pathway,” “cell surface receptor protein serine/threonine kinase signaling pathway,” “response to transforming growth factor beta,” and “cellular response to transforming growth factor beta stimulus.” Notably, this pattern reflects enrichment not only for components of the signaling pathway itself, but also for genes associated with cellular responses to this signaling axis. The presence of “mitotic cell cycle phase transition” among the top 10 is also consistent with the highest proliferative activity of SC-Lhx9-13.5 among supporting lineage clusters at this stage (Figure 8A). On E16.5, SC-Lhx9 showed strong representation of extracellular organization terms, with “extracellular matrix organization,” “extracellular structure organization,” and “external encapsulating structure organization” all present in the top 10. At the same time, TGFβ-related terms, specifically, “response to transforming growth factor beta” and “cellular response to transforming growth factor beta stimulus,” remained enriched, indicating sustained TGFβ-response at the later stage together with pronounced ECM organization programs (Figure 10A).

Although signaling-related terms did not always appear within the top 10 upregulated categories for every cluster, their enrichment was statistically significant across all comparisons. Specifically, we consistently detected significant enrichment of key signaling-associated GO terms, including “Wnt signaling pathway,” “transforming growth factor beta receptor superfamily signaling pathway,” “cell surface receptor protein serine/threonine kinase signaling pathway,” “response to transforming growth factor beta,” and “cellular response to transforming growth factor beta stimulus.” Moreover, all clusters showed significant enrichment of the term “response to BMP” and “cell-cell adhesion via plasma-membrane adhesion molecules.” In addition, “Notch signaling pathway” and “cellular response to fibroblast growth factor stimulus” emerged among the enriched categories for the SC-Lhx9 sub-clusters (Figure 10A).

For preSC5 on E11.5, the top 10 downregulated GO terms (Figure 10B) were entirely represented by metabolism-related categories, including “canonical glycolysis,” “glucose catabolic process to pyruvate,” “glucose catabolic process,” “glycolytic process through glucose-6-phosphate,” “glycolytic process through fructose-6-phosphate,” as well as “NADH regeneration,” “NAD catabolic process,” and “NAD metabolic process.” This distribution indicates that energy and glycolytic processes dominate the downregulated programs in preSC5. In contrast, for SC-Lhx9-12.5, the top 10 downregulated categories simultaneously included terms related to male gonad development and differentiation: “male gonad development,” “sex determination,” “male sex differentiation,” and “development of primary male sexual characteristics,” together with energy-related processes such as “oxidative phosphorylation,” “aerobic respiration,” and “generation of precursor metabolites and energy.” At later stages, SC-Lhx9-13.5 and SC-Lhx9-16.5, the top 10 downregulated terms were dominated by “oxidative phosphorylation,” “aerobic respiration,” “cellular respiration,” “generation of precursor metabolites and energy,” and a set of terms reflecting ATP synthesis and electron transport processes: “ATP biosynthetic process,” “ATP synthesis coupled electron transport,” “mitochondrial ATP synthesis coupled electron transport,” “proton motive force-driven mitochondrial ATP synthesis,” and “proton motive force-driven ATP synthesis,” with “aerobic electron transport chain” also present on E16.5. Collectively, these data show that downregulated gene sets in SC-Lhx9 are enriched for oxidative phosphorylation, respiration, ATP synthesis, and male gonad development programs, whereas preSC5-11.5 is characterized by reduced glycolytic and NAD/NADH-related processes (Figure 10B).

### 3.10 Intercellular communication landscape of the supporting cell lineage in the embryonic testis

**FIGURE 11:**
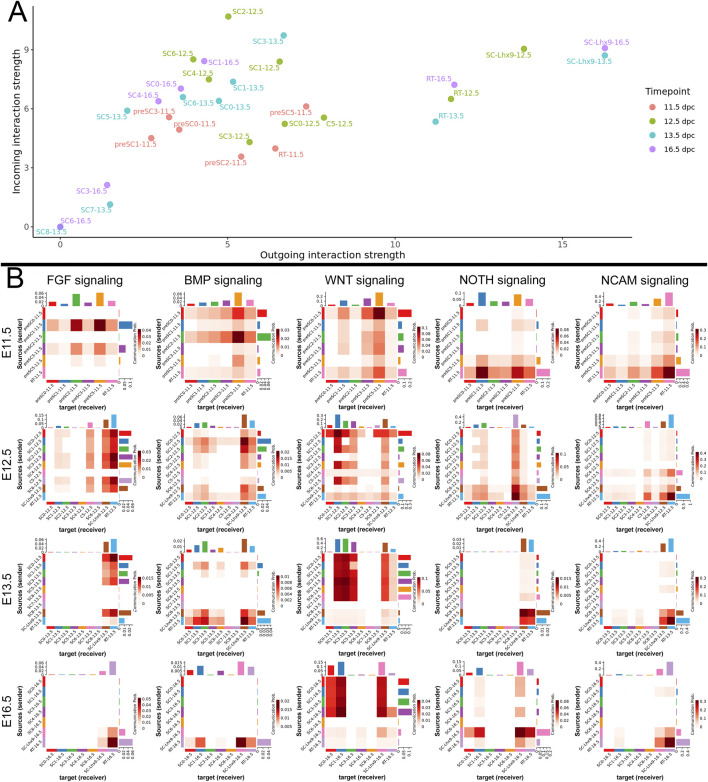
CellChat analysis of intercellular communication in the embryonic testis. **(A)** A scatterplot of cumulative outgoing (x-axis) versus incoming (y-axis) interaction strength for the preSC, C5, SC, SC-Lhx9, and RT sub-clusters. **(B)** Heatmaps for five signaling pathways depicting the interaction probability between sender (rows) and receiver (columns) cell types at each stage. The color intensity reflects the inferred likelihood of a given ligand-receptor interaction. The bars above and to the right of each heatmap indicate the cumulative interaction strength of each sub-cluster as a receiver and as a sender, respectively.

We inferred intercellular communication with CellChat (v1.6.1) using the single-cell RNA-seq dataset of Mayère et al. (2022). CellChat is an R package that reconstructs cell-cell signaling networks from single-cell transcriptomic data by using a manually curated ligand-receptor interaction database (Jin et al., 2021). We summarized the inferred networks at the ligand-receptor and pathway levels to identify the main signaling routes and the sender/receiver cell populations across the preSC, C5, SC, SC-Lhx9, and RT sub-clusters (E11.5-E16.5) (Figure 5A). Across the analyzed stages, the SC-Lhx9 sub-clusters consistently emerged as prominent signaling hubs, exhibiting high incoming and outgoing interaction strength, whereas RT cells showed a marked increase in outgoing signaling strength after E11.5 (Figure 11A).

To link GO-defined biological programs with CellChat-inferred intercellular interactions, we cross-referenced the GO-ORA enrichment profile (Figure 10) with CellChat pathway-level predictions. Five pathways, specifically FGF, BMP, WNT, NOTCH, and NCAM, were supported by both analyses. Although no NCAM-specific GO term emerged, enrichment of “extracellular matrix organization” and “cell-cell adhesion via plasma-membrane adhesion molecules” was consistent with NCAM-mediated adhesion and signaling.

#### 3.10.1 FGF pathway

The CellChat analysis showed that FGF ligands were predominantly secreted by the preSC1 and preSC3 clusters on E11.5, whereas the receivers comprised virtually all clusters (Figure 11B). On E12.5 and E13.5, the main sources of FGF signaling included various SC populations as well as SC-Lhx9 cells, whereas the primary receivers were SC-Lhx9, C5, and RT cells. By E16.5, FGF signaling occurred predominantly among RT and SC-Lhx9 cells (Figure 11B). At all the analyzed stages, the top 10 putative ligand-receptor pairs included *Fgf9*-*Fgfr1* or *Fgf9*-*Fgfr2* with RT, preSC, and SC-Lhx9 acting as receivers (Supplementary Material 3).

#### 3.10.2 BMP pathway

PreSC5-11.5 and SC-Lhx9-12.5, 13.5, and 16.5 were the primary target clusters for BMP signaling. We also detected a progressive increase in outgoing BMP signals from RT between E11.5 and E16.5 (Figure 11B). On E11.5, the top 10 putative ligand-receptor pairs included *Bmp2*-(*Bmpr1a* + *Bmpr2*), with preSC and RT as receivers. On E13.5 and E16.5, the top 10 included *Bmp4*-(*Bmpr1a* + *Bmpr2*), with RT as a sender and RT and SC-Lhx9 as receivers (Supplementary Material 3).

#### 3.10.3 WNT pathway

The CellChat analysis indicated that preSC5-11.5 was the main signal receiver for WNT signaling on E11.5. At this stage, the RT was one of the two most prominent WNT signal sources, and the top 10 ligand-receptor interactions included *Wnt4*-(*Fzd3*+*Lrp6*) with the RT as the sender. For later stages, SC clusters were the dominant source and targets of WNT signaling. Across all stages (E11.5-E16.5), *Wnt6*-(*Fzd3*+*Lrp6*) or *Wnt6*-(*Fzd2*+*Lrp6*) remained in the top 10 for both preSC and SC-Lhx9 (Figure 11B, Supplementary Material 3).

#### 3.10.4 NOTCH pathway

On E11.5, the RT was the main NOTCH sender, whereas preSC1-11.5 and preSC5-11.5 were the primary receivers (Figure 11B). On E12.5-E16.5, the predicted NOTCH interactions were enriched for interactions involving the RT and SC-Lhx9 (Figure 11B). On E12.5, RT-12.5 and SC-Lhx9-12.5 emerged as the dominant senders, primarily targeting SC6-12.5, RT-12.5, and SC-Lhx9-12.5. Notably, *Dlk1*-(*Notch2*) was the leading ligand-receptor pair both on E11.5 and E12.5, with the RT as a sender and preSC and SC as receivers. On E13.5, NOTCH signaling was dominated by *Dlk1*-(*Notch3*), with top interactions detected for SC-Lhx9/RT, RT/SC-Lhx9, and RT/RT. By E16.5, SC-Lhx9-16.5 became the principal NOTCH sender, with the top pairs *Dlk1*-(*Notch2*) and *Dlk1*-(*Notch1*), targeting SC-Lhx9 and the RT as receivers (Figure 11B, Supplementary Material 3). This pattern is consistent with the GO-ORA results, which show enrichment of NOTCH signaling at the E13.5 and E16.5 stages in SC-Lhx9 (Figure 10A).

#### 3.10.5 NCAM pathway

On E11.5, RT-11.5 showed autocrine NCAM signaling and also formed interactions with preSC5-11.5 (Figure 11B). The top 10 interactions were dominated by *Ncam1*-(*Ncam1*), with RT-11.5 acting as both a sender and a receiver and with reciprocal links between RT-11.5 and preSC5-11.5. The top 10 also included *Ncam1*-(*Fgfr1*), with RT-11.5 as a sender and preSC5-11.5 as a receiver (Supplementary Material 3). By E12.5, the newly emergent C5-12.5 cluster and SC-Lhx9-12.5 both interacted with RT-12.5, and the top 10 interactions were again dominated by *Ncam1*-(*Ncam1*). In addition, *Ncam1*-(*Fgfr1*) was present among the top 10, with RT-12.5 as a sender and SC-Lhx9-12.5 as a receiver (Supplementary Material 3). On E13.5 and E16.5, SC-Lhx9 and the RT remained the principal NCAM-connected populations (Figure 11B), and at both stages, the top 10 interactions were dominated by *Ncam1*-(*Ncam1*), with the RT and SC-Lhx9 represented as both senders and receivers, and consistently included *Ncam1*-(*Fgfr1*) with the RT and/or SC-Lhx9 as sender/receiver partners (Figure 11B; Supplementary Material 3).

Taken together, the CellChat results corroborate the GO-ORA findings and reveal a distinct signaling signature involving the FGF, BMP, WNT, NOTCH, and NCAM pathways that distinguishes the preSC5 and SC-Lhx9 sub-clusters from the remaining SC populations during the time of RT formation in embryonic development.

## 4 Discussion

In this study, we characterized the embryonic development of the mouse RT by integrating chimeric organotypic UGC cultures, reanalysis of testis scRNA-seq data from Mayère et al. (2022), including RNA velocity and inferred intercellular communication, and quantitative assessment of RT cell proliferation. This combined framework provided evidence consistent with the possibility that a subset of RT cells may originate from the SC lineage and to propose a model in which the distal RT segment promotes formation of the proximal RT compartment adjacent to the testis cords. This interpretation is consistent with prior *in vivo* observations that cells at the RT-testis cord interface co-express markers of both SC and RT cell lineages (Kulibin and Malolina, 2020).

### 4.1 Proximal RT cells may derive from the SC lineage

Organotypic culture of embryonic gonads and chimeric UGC assembly have been widely used to resolve the origins of testicular somatic cell populations, testis cord morphogenesis, and mesonephric cell migration (Martineau et al., 1997; Capel and Batchvarov, 2008; Imaimatsu et al., 2023). In most studies, however, the RT was not considered as the primary object of analysis, but was used as an anatomical landmark structure for other experimental aims instead (Imaimatsu et al., 2023).

In this study, we have demonstrated that RT morphogenesis in organotypic culture of intact UGCs recapitulates key topographic and marker-defined features observed *in vivo*, with the expected developmental delay in culture (Figures 1A–C). These results support the use of intact UGC culture as a reliable and experimentally tractable model of embryonic RT development. We have also generated chimeric organ cultures with differentially labelled gonadal and mesonephric tissues (GFP^+^/GFP^−^), which has allowed us to directly track their contributions to the forming RT (Figures 3A–H). In these chimeras, after 72 h, we detected PAX8^+^ cells within the gonadal compartment, including a subset of PAX8^+^/AMH^+^ cells located inside testis cords (Figures 3D–H).

The spatial relationship between PAX8^+^ structures arising within the gonadal compartment and the distal RT in the mesonephric compartment varied. In some samples, PAX8^+^ cells formed continuous domains that appeared to connect with the mesonephric RT (Figure 3F). In other samples, newly formed PAX8^+^ domains, including both PAX8^+^/AMH^+^ and PAX8^+^/AMH^−^ cells, were located at a substantial distance from the distal RT (Figure 3D). One plausible explanation is that chimera assembly disrupts the native RT-cord topology, displacing gonadal regions that would normally be adjacent to the RT. At the same time, the formation of RT-like PAX8^+^ domains, even when they are spatially separated from the distal RT, suggests that, by E12.5, the gonadal subset competent to generate RT cells may already be primed toward an RT fate. Under these experimental conditions, such primed cells may initiate RT-like morphogenesis irrespective of their final position within the reconstructed UGC.

Together, these observations indicate that gonadal cells can generate PAX8^+^ RT-like structures in the chimeric setting. Moreover, the presence of PAX8^+^/AMH^+^ cells specifically within the testis cords suggests that a proportion of cord-associated PAX8^+^ cells arises from the SC lineage, potentially via an intermediate PAX8^+^/AMH^+^ state. This interpretation aligns with morphogenetic reconstructions showing that the RT develops at the testis-mesonephros border through remodeling of testis cord regions (Combes et al., 2009; Nel-Themaat et al., 2009). It is also consistent with observations of cells co-expressing SC and RT markers at the RT-cord interface (Kulibin and Malolina, 2020). In line with this possibility, Rebourcet et al. (2014) crossed *Amh*-Cre mice with a Cre-inducible YFP reporter and detected *Amh*-Cre lineage labeling in a subset of RT cells in 17-day-old mice, which supports the presence of an RT subpopulation derived from *Amh*-expressing cells.

A methodological limitation of UGC-based lineage contribution analyses is their sensitivity to dissection precision, as inadvertent inclusion of adjacent structures can introduce artefacts, including the transfer of pre-existing mesonephric PAX8^+^ cells into the gonad. Although occasional dissection-related contamination cannot be fully excluded, the rare detection of PAX8^+^ cells in cultured testis controls, compared with their reproducible presence in chimeric UGCs, makes a dissection artefact explanation unlikely. Thus, the observed contribution is best explained by a biological effect rather than technical contamination.

### 4.2 RNA velocity supports a transient SC-to-RT transition during embryogenesis

To corroborate our chimera-based observations using an independent *in silico* approach, we reanalyzed published testis single-cell RNA-seq data from Mayère et al. (2022), with a specific focus on the putative SC-RT axis. To maximize the resolution along this axis, where rare and transient intermediates are expected, we reclustered only preSC, SC, and RT populations. This focused strategy reduces masking effects from unrelated gonadal cell types present in global clustering and enables a more stable identification of transitional states. Consistent with this rationale, prior work has suggested transitional states between the SC lineage and RT cells (Mayère et al., 2022).

We then applied RNA velocity, which predicts the directions of cell state transitions based on unspliced-to-spliced transcript ratios (La Manno et al., 2018). In line with our expectations, RNA velocity analysis of the Mayère et al. (2022) dataset indicated that the inferred transition towards the RT was most prominent in clusters exhibiting mixed marker profiles that were resolved by the targeted reclustering, including SC-Lhx9-E12.5, SC-Lhx9-E13.5, and C5 (Figures 6A–C). By E16.5, this velocity signal largely disappeared. In particular, C5 showed trajectories towards both the RT and SC-Lhx9 on E12.5. Taken together, these results support an early, time-restricted SC-RT transition.

A broadly similar dynamic was reported by Kay et al. (2024), where RNA velocity trajectories were described from preSCs towards *Pax8*-expressing cells, corresponding to the RT lineage. Mayère et al. (2022) reported transcriptomic similarity between *Pax8* ^+^ supporting-like cells and SC lineage populations and demonstrated *Pax8* ^+^ cell lineage contribution to RT structures; their PAGA reconstruction resolved Sertoli like cell and SC as distinct coelomic epithelium-derived trajectories.

### 4.3 Candidate intermediate states: C5 and SC-Lhx9 clusters

Based on our reclustering analysis and RNA velocity inference, we identified two candidate intermediate states along the RT-forming trajectories: the C5 and SC-Lhx9 clusters. C5 expressed SC markers, including *Sox9* and *Sf1*, together with RT-associated extracellular matrix/adhesion genes, including *Ncam1* (Møller et al., 1991), *perlecan* (*Hspg2*) (Miqueloto and Zorn, 2007), and *biglycan* (*Bgn*) (Figure 5E). In contrast, *Amh* and *Pax8* were not detected in this cluster. C5 also expressed *Lhx9*, a marker of undifferentiated gonadal somatic cells (Mazaud et al., 2002) (Figures 5B,E).

C5 was resolved on E12.5, but did not emerge as a distinct cluster on E13.5 or E16.5 (Figures 5B–E). Immunohistochemistry indicated that cells with a corresponding marker profile were abundant on E12.5, but became less frequent by E13.5 (Figure 7B). Spatially, a subset of these cells was located close to the RT. Taken together, these observations are consistent with C5 representing an immature embryonic progenitor state capable of contributing to both the RT and SC-Lhx9 trajectories.

A second notable cluster, SC-Lhx9, comprised supporting cells with elevated *Lhx9* transcription factor expression against the background of canonical SC markers (Figures 5B–E). *Lhx9* expression is normally the highest during early gonad formation and decreases after SC differentiation (Mazaud et al., 2002). Here, we identified a SC subpopulation that retains *Lhx9* expression from E12.5 to E16.5, potentially reflecting delayed differentiation or partial reactivation of an early developmental program. SC-Lhx9 also displayed a mixed phenotype through the expression of RT marker matrix genes (Miqueloto and Zorn, 2007) such as *Dcn*, *Bgn, Hspg2*, and *Ncam1* (Figure 5E). The presence of this cluster in E12.5 testes was supported by immunofluorescence. PDGFRA-positive cells corresponding to this cluster were located within testis cords adjacent to the RT (Figure 7A). Taken together, these findings raise the possibility that the PAX8^+^/AMH^+^ cells observed in the chimeric cultures arise from the SC-Lhx9 cluster.

Taken together, bioinformatics inference, chimera experiments, and the spatial positioning of newly arising RT-like cells in chimeric UGCs suggest that after E12.5, new RT cells likely arise from a pre-existing pool of progenitors belonging to the SC lineage.

### 4.4 Contribution of RT cell proliferation to the increase in RT cell numbers

An important and still unresolved aspect of RT morphogenesis is the cellular basis of its expansion, which may be driven predominantly either by the proliferation of resident RT cells or by a continuous recruitment of additional cells from external sources. Our data show that proliferative activity within the RT remains low through E15.5 (Figures 8A,B). A similar phenomenon was reported by Mayère et al. (2022), where RT precursors displayed weak mitotic activity following sex determination. It was not until E16.5 that we observed a sharp increase in mitotic activity among RT cells. In contrast, the surrounding SCs within testis cords continue to proliferate robustly over these stages (Archambeault and Yao, 2010; Miyabayashi et al., 2013).

By combining our measurements of RT proliferative dynamics with estimates of total RT cell numbers, we modeled the extent to which local proliferation could account for the increase in RT population size (Figures 8B,C). We first analyzed the available literature and found no direct estimates of cell-cycle length for RT cells or SCs at the embryonic stages examined in this study. Because our experimental data suggested that local proliferation of RT cells might be insufficient to explain the observed dynamics of RT population growth, we intentionally used the most permissive assumptions for a proliferation-based model. Specifically, we applied a minimal cell-cycle length estimate of 12 h, derived from highly proliferative neural progenitors in the embryonic cortical germinal zone (Mi et al., 2018). This value was used as a lower-bound estimate to calculate the maximum RT cell numbers that could be expected if expansion were driven exclusively by proliferation. Because longer cell-cycle estimates would only decrease the predicted contribution of local proliferation, the use of a 12 h lower-bound estimate provides the most conservative test of whether proliferation alone could account for RT expansion. Even under this optimistic assumption, proliferation alone could not explain the observed early increase in the RT population: the fraction of dividing RT cells was too small to support population expansion without the recruitment of additional cells.

These data are consistent with the possibility that new cells are recruited into the RT from outside sources, potentially including the SC lineage. Notably, cluster-resolved proliferation analysis within the supporting cell lineage indicates that the highest proliferative activity on E12.5-E13.5 occurs in the C5 and SC-Lhx9 clusters (Figure 8A), the same clusters for which a transition towards the RT cluster is inferred by RNA velocity (Figure 6). This concordance provides additional, indirect support for our model of proximal RT origin.

Finally, from E14.5 onwards, RT proliferation becomes predominantly concentrated in the proximal region. This spatial bias is in line with a contribution of SCs and/or other gonadal cell types to the proliferative activation of RT cells on E16.5.

### 4.5 The distal RT promotes the formation of proximal PAX8^+^ cells

By varying dissection conditions, we tested whether the amount of distal RT retained within the mesonephric compartment influenced the formation of the proximal RT structures within the gonad. Across two experimental series differing in the extent of distal RT preservation, retention of a larger distal RT segment was associated with increased numbers of PAX8^+^ cells forming in the gonadal compartment (Figure 3). Notably, the proportion of PAX8^+^/AMH^+^ cells among all gonadal PAX8^+^ cells was comparable between the experimental series (Figure 3I), suggesting a shared mechanism of PAX8^+^ cell emergence across all the studied conditions. These results support a model in which the distal RT segment acts as an inducer for proximal RT components: signals from already established distal elements may stimulate SC lineage cells or upstream progenitors to initiate *Pax8* expression and adopt an RT program.

A similar principle has been proposed for the postnatal testis. Uchida et al. (2022) reported that disruption of *Sox17* in the RT epithelium impaired SC valve formation at the border between the RT and seminiferous tubules, whereas *Sox17*-cKO SCs retained the capacity to undergo this process when exposed to an environment containing an intact SOX17^+^ RT (*Amh*-Treck transplantation). Consistent with these functional data, the authors’ scRNA-seq analysis, together with *in situ* hybridization results, supported the view that the RT epithelium can act as a local paracrine signaling center whose secreted factors influence neighboring SCs.

### 4.6 Intercellular signaling: CellChat and GO-ORA

Our chimera experiments support an inductive model of RT morphogenesis in which an already established distal RT can promote the emergence of the proximal RT through paracrine signaling. Here, we therefore analyze and discuss which signaling pathways may mediate that process and RT-SC communication.

Inference of intercellular communication using CellChat, together with functional gene annotation (GO-ORA), highlighted a set of signaling pathways implicated in mouse RT development (Figures 10A, 11B). Both analyses converged on canonical morphogenetic programs, including the FGF, BMP, WNT, and NOTCH pathways linked to somatic differentiation. In addition, NCAM-mediated signaling was indirectly represented in GO-ORA (term “cell-cell adhesion via plasma-membrane adhesion molecules”) and emerged as significant in CellChat (Figures 10A, 11B). Below, we interpret these pathways in the context of our data. Specifically, we infer putative sending and receiving cell populations for each signaling pathway (ligand-receptor interaction set) and relate these patterns to established models of RT morphogenesis.

CellChat indicates that on E11.5-E13.5, the SC and preSC clusters are the predominant sources of FGF ligands, whereas C5, RT, preSC5, and SC-Lhx9 express FGF receptors and thus represent putative receivers. This configuration aligns with the established role of *Fgf9* downstream of *Sry* in supporting key testis programs, including SC differentiation and proliferation, gonadal mesenchyme proliferation, and mesonephric migration (Colvin et al., 2001; Schmahl et al., 2004; Kim et al., 2007). Notably, across all the stages examined, the RT emerges as a particularly FGF + compartment: the dominant inferred interaction at each time point is *Fgf9*-*Fgfr2* (Supplementary Material 3). Up to E16.5, this incoming signal is inferred to originate primarily from neighboring SCs. On E16.5, however, the RT is inferred to function as both sender and receiver, a switch that may relate to the abrupt rise in RT mitotic activity observed at this stage. The SC-Lhx9-16.5 sub-cluster also continues to contribute to the FGF ligand pool (Figure 11B). Supplementation with FGF9 was reported to accelerate the emergence of RT-like structures in a testis regeneration model (Awang-Junaidi et al., 2022a; Awang-Junaidi et al., 2022b). Taken together, these observations support the notion that SCs promote RT epithelial maturation and proliferation via stage-dependent paracrine cues.

BMP pathway activity connected the RT, SC-Lhx9, and preSC5 clusters. In particular, the RT cluster showed increasing BMP signaling output across E11.5-E16.5, predominantly targeting the preSC5 and SC-Lhx9 clusters (Figure 11B). *Bmp4* emerged as a key mediator of this network: *Bmp4* expressed in the RT was predicted to act autocrinally on the RT and paracrinally on the preSC5 and SC-Lhx9 clusters via *Bmpr1a*/*Bmpr2* receptors. This finding is in line with previous report of *Bmp4* expression in the RT epithelium (Hu et al., 2004; Imura-Kishi et al., 2021).

NOTCH signaling in the fetal testis has been shown to maintain interstitial cells in a progenitor-like state and thereby restrain fetal Leydig cell differentiation (Tang et al., 2008), underscoring the broader role of this type of signaling in balancing differentiation versus progenitor maintenance. In our dataset, CellChat points to active NOTCH communication between SC-Lhx9 and the RT on E13.5-E16.5. This pattern is compatible with NOTCH acting as a short-range, contact-dependent signal at the cord-RT interface that helps to maintain a clear boundary between cord-resident SC-lineage programs and RT-associated programs. In an RT morphogenesis context, such signaling could help determine which cells remain within cords and which adopt an RT-associated program. We therefore propose that NOTCH functions here as a local regulator of somatic fate dynamics, potentially delaying the maturation of a subset of SC-lineage cells and permitting entry into a transient hybrid state compatible with incorporation into the RT.

According to classical immunohistochemistry, the RT becomes NCAM1+ by E11.5 and remains NCAM1+ throughout development and into adulthood (Møller et al., 1991). After the segregation and maturation of the Sertoli lineage, differentiated SCs within testis cords are largely NCAM1− (Møller et al., 1991; Sharpe et al., 2003; Pauls et al., 2006; Yang and Han, 2010). *In situ* hybridization and immunohistochemistry further reported a continuity of NCAM1+ cells between the RT region and testis cords (Mayerhofer et al., 1996), consistent with the presence of an NCAM1+ interface zone at the RT-testis cord boundary where cells may exhibit mixed or intermediate features.

In our CellChat analysis, NCAM-mediated signaling is highly specific to the RT and SC-Lhx9. We have detected *Ncam1*-*Fgfr1* interactions in which the RT acts as a sender and SC-Lhx9 acts as a receiver across E11.5-E16.5 (Supplementary Material 3). NCAM1 was reported to stabilize FGFR1 on a neighboring cell promoting cell migration (Francavilla et al., 2009). A similar mechanism might underlie testis cord disassembly and reassembly in the RT-adjacent region (Combes et al., 2009; Nel-Themaat et al., 2009; Kulibin and Malolina, 2020), as well as stabilization of connectivity between the RT and testis cords.

Another ECM-related factor is perlecan encoded by *Hspg2*, which is expressed not only in the RT but also in preSC5, C5, and SC-Lhx9 (Figure 5E). Miqueloto and Zorn (2007) report that in mice, perlecan is detectable in the basement membranes of developing testis cords already on E13-E15. By E14-E15, the testis cords, including those segments that are morphologically connected to the proximal RT, are surrounded by perlecan-rich basement membranes, highlighting a spatial link between perlecan deposition and the testis cord-RT interface (Miqueloto and Zorn, 2007).

*Hspg2* is a basement membrane heparan sulphate proteoglycan that, by general principles, can modulate proliferative responses through binding, sequestration, and presentation of growth factors and morphogens (Hayes et al., 2022). This is particularly relevant for FGF signaling, as heparan sulphate proteoglycans have long been shown to regulate FGF-FGFR interactions and signaling output (Yayon et al., 1991; Ornitz et al., 1992). This is of interest in the present context because FGF signaling, specifically FGF9 and FGF2, promotes the proliferation of SC precursors and immature SCs in culture (Van Dissel-Emiliani et al., 1996; Schmahl et al., 2004; Kim et al., 2007). Moreover, perlecan has been reported to bind and present FGF2 and FGF9 (Knox et al., 2002; Melrose et al., 2006).

Overall, a view of perlecan as a local presenter of morphogens, particularly of FGF cues, offers a mechanistic framework that could plausibly contribute to the elevated proliferative activity of SC-Lhx9 relative to other supporting lineage clusters. This interpretation is also compatible with our observation that RT proliferation becomes concentrated in the proximal region at later stages (Figures 9D–F). Importantly, however, the effects of perlecan are strongly context-dependent and its roles can be opposing across cell types and tissue states, which may help explain why perlecan is present in the RT, yet proliferative activity remains low in the distal RT (Hayes et al., 2022).

In our analysis, WNT signaling emerges as a key regulator of early gonadal somatic programs and RT morphogenesis. Within the SC lineage, we specifically highlight the potential relevance of WNT6-associated signaling: *Wnt6*/WNT6 expression has been reported in pre-SCs on E11.5 and in SCs throughout embryogenesis in mice (Cory et al., 2007; Garcia-Alonso et al., 2022), as well as being used as an SC marker in the human gonadal development atlas (Garcia-Alonso et al., 2022). For the RT at the early stages (E11.5-E13.5), we have detected a prominent RT-derived WNT4 signal, with SC and SC-Lhx9 emerging as putative signal recipients. Notably, the importance of *Wnt4* expression for RT development has been recently demonstrated: in XY embryos, *Wnt4* knockout results in a markedly reduced or absent RT, accompanied by a disrupted organization of adjacent testis cords (Mayère et al., 2022). More broadly, these observations align with current models suggesting a multi-layered involvement of the WNT pathway in testis development and somatic lineage regulation (Dong et al., 2015).

Together, our data provide several lines of evidence supporting the contribution of SC-lineage cells to a subset of RT cells during embryonic development. Our mathematical modeling suggests that expansion of the RT cell population cannot be explained by proliferation alone and may also involve the recruitment of new cells from external sources. Using chimeric UGC assays, we obtained evidence supporting the possibility that a subset of RT cells can arise from SCs and early supporting progenitor cells preserved in the C5 gonadal compartment. However, because this approach critically depends on the accuracy of tissue dissection, we statistically evaluated the possibility that these results could be explained by experimental error and found this explanation to be highly unlikely. At the same time, the chimeric UGC assay identifies the anatomical source of cells contributing to the proximal RT, but does not by itself determine their lineage identity. The SC-lineage origin of these cells is therefore supported by additional evidence, including immunohistochemical detection of SC markers in RT cells formed in chimeric UGC cultures and bioinformatic modeling of differentiation trajectories using RNA velocity analysis. Nevertheless, definitive confirmation of this lineage relationship will require future lineage-tracing experiments. Moreover, although our data indicate the emergence of gonad-derived Pax8^+^ cells with RT-associated features, they do not demonstrate that these cells ultimately persist and differentiate into functional RT cells in the adult testis. In addition, our data suggest that the distal RT segment may exert an inductive influence, promoting *Pax8* expression in neighboring supporting-lineage cells and thereby facilitating the formation of the proximal RT compartment. Consistent with this interpretation, by E12.5, a pool of RT-competent progenitors is likely already present and may represent the target for this distal RT-derived signal. By integrating organotypic culture with single-cell transcriptomic analyses, we identified molecular pathways that may coordinate interactions between the emerging RT and SC-lineage populations, including FGF, WNT, BMP, and NOTCH signaling, as well as NCAM-mediated programs. Together, these findings support a working model of RT morphogenesis in which a subset of SC-lineage cells may undergo fate conversion and integrate into the RT under the influence of signals produced by previously established RT structures. This framework advances our understanding of somatic lineage plasticity during testis development and provides a foundation for future studies of the regulatory mechanisms governing the differentiation of SC-lineage cells into RT cells and the cross-talk between SCs and the RT.

## Data Availability

Publicly available datasets were analyzed in this study. This data can be found here: https://www.ncbi.nlm.nih.gov/geo/query/acc.cgi?acc=GSE184708 NCBI GEO, GSE184708.
