# Supplementary material for: Evidence of Sertoli cell lineage contribution to the rete testis population during embryonic development
Source: Front Cell Dev Biol. 2026 Jun 5;14:1849381. doi: 10.3389/fcell.2026.1849381 (PMC13278955; doi:10.3389/fcell.2026.1849381)
Supplement: Supplementary file 1 [file Supplementaryfile1.docx]

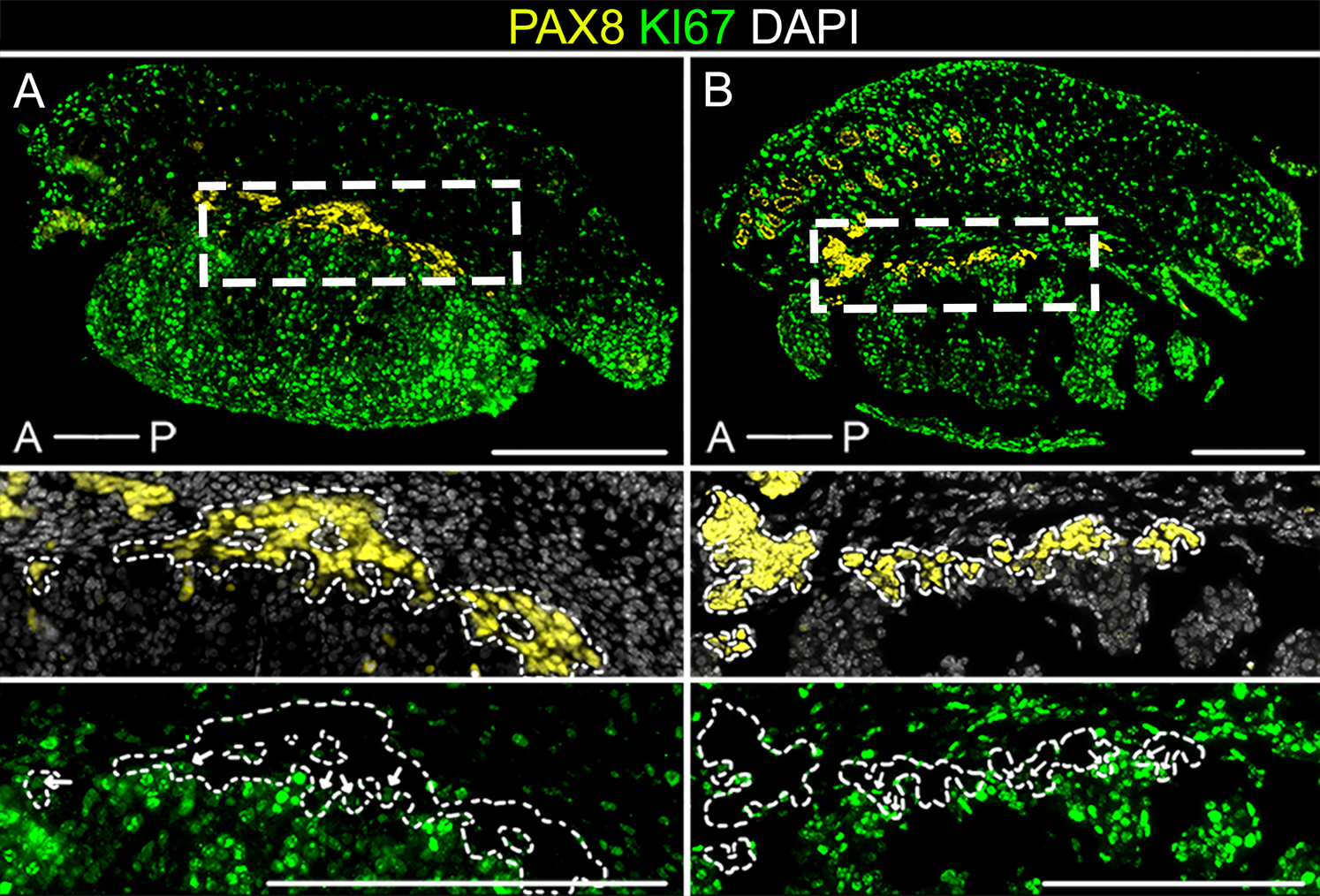


**Supplementary Figure 1.** **The** **distribution of PAX8⁺/KI-67⁺ cells in the RT on E13.5 and E15.5.** (A-B) Representative images of the sections on E13.5 (A) and E15.5 (B), stained for PAX8 and KI-67. The bottom panels show magnified images of the boxed areas from (A-B); the arrows indicate PAX8⁺/KI-67⁺ RT cells; and the dashed outline delineates the RT. The panels are composites stitched from several adjacent fields of view. Scale bars: (A-B), 200 µm.
